# Spectroscopic monitoring of transport processes during loading of ovarian tissue with cryoprotective solutions

**DOI:** 10.1038/s41598-019-51903-5

**Published:** 2019-10-30

**Authors:** Jiale Han, Bulat Sydykov, Huaqing Yang, Harald Sieme, Harriëtte Oldenhof, Willem F. Wolkers

**Affiliations:** 1 Unit for Reproductive Medicine - Clinic for Horses, and Biostabilization Laboratory - Lower Saxony Centre for Biomedical Engineering, Implant Research and Development, University of Veterinary Medicine Hannover, Hannover, Germany; 2 Present Address: BioNTech, Mainz, Germany

**Keywords:** Permeation and transport, Biomedical engineering, Translational research

## Abstract

There is an increasing demand for female fertility preservation. Cryopreservation of ovarian cortex tissue by means of vitrification can be done ad-hoc and for pre-pubertal individuals. Obtaining a homogeneous distribution of protective agents in tissues is one of the major hurdles for successful preservation. Therefore, to rationally design vitrification strategies for tissues, it is needed to determine permeation kinetics of cryoprotective agents; to ensure homogeneous distribution while minimizing exposure time and toxicity effects. In this study, Fourier transform infrared spectroscopy (FTIR) was used to monitor diffusion of different components into porcine ovarian cortex tissue. Water fluxes and permeation kinetics of dimethyl sulfoxide (DMSO), glycerol (GLY), ethylene glycol (EG), and propylene glycol (PG) were investigated. Diffusion coefficients derived from FTIR data, were corroborated with differential scanning calorimetry and osmometer measurements. FTIR allowed real-time spectral fingerprinting of tissue during loading with mixtures of protective agents, while discriminating between different components and water. Exposure to vitrification solutions was found to cause drastic initial weight losses, which could be correlated with spectral features. Use of heavy water allowed distinguishing water fluxes associated with dehydration and permeation, both of which were found to precede permeation of cryoprotective agents. Overall, DMSO and EG were found to permeate faster than GLY and PG. In mixtures, however, solutes behave differently. The non-invasive spectroscopic method described here to study permeation of vitrification solution components into ovarian tissue can be applied to many other types of engineered constructs, tissues, and possibly organs.

## Introduction

In human reproductive medicine, there is an increasing demand for female fertility preservation for cancer patients as well as for personal reasons. Options for fertility preservation include cryopreservation of embryos and oocytes, as well as ovarian tissue^1–3^. Ovarian tissue cryopreservation has the advantage that it can be performed on short notice, irrespective of the cycle stage, and it even is an option for pre-pubertal individuals. If preserved ovarian tissue is transplanted, e.g. after anti-cancer treatment, hormonal functions and fertility can be restored^4–7^. In addition, tissue containing various follicle stages can be used for cultivation and retrieval of oocytes^8–10^, for use in *in vitro* fertilization procedures. The latter is especially interesting for domestic and endangered animal genetic reserves^11^.

Ovarian tissue cryopreservation can be done using either slow-freezing or vitrification^12,13^. In both cases, cryoprotective agents (CPAs) need to be added to minimize the damaging effects of cooling and/or ice formation. With slow-freezing, low or moderate concentrations of permeating CPAs, like dimethyl sulfoxide or glycerol, are used to minimize the damaging effects of ice formation. Here it is important to find the optimal cooling rate, since it directs the timing and location of ice formation, solute concentration in the liquid phase, and therewith the extent of mechanical and osmotic stress cells are exposed to^14–16^. The optimal cooling rate with maximal cryosurvival is dependent on cell specific membrane permeability characteristics for water and protective agents, which is more complex in case of tissues containing different cell types and the connective matrix forming a diffusion barrier for CPAs. In addition, the medium composition, solute concentrations, temperature and presence of ice affect cell membrane permeability characteristics^17^.

The damaging effects of ice formation can be avoided using vitrification or ice-free cryopreservation, which is typically preferred in case of tissues. Vitrification is done using high CPA concentrations and high cooling rates, allowing specimens to directly turn into an amorphous state protecting embedded structures while arresting reactions^18,19^. Typically, mixtures of permeating agents and step-wise CPA loading and removal protocols are used to reduce CPA toxicity and remain within the osmotic tolerance limits^20^. Vitrification of larger tissues is especially challenging due to limitations in homogeneous CPA mass transfer and inhomogeneous heat transfer during rapid cooling and warming. Application of very high CPA concentrations allows for use of lower cooling rates. However, this causes a decline in cell viability already prior to cooling due to CPA toxicity^21^.

To rationally design cryopreservation protocols for tissue vitrification, insights in permeation kinetics of CPAs into tissues are needed to ensure maximum permeation and homogeneous distribution of CPAs while minimizing the exposure time and toxicity effects. Various methods have been applied to study CPA permeation in tissues, including nuclear magnetic resonance^22,23^ and X-ray computer tomography^24,25^. Furthermore, osmometer measurements of solutions in which CPA-permeated tissue was equilibrated have been employed to derive CPA diffusion coefficients^26,27^. Most of these approaches, however, cannot be used to discriminate between the different components in vitrification solutions. This can be done using Fourier transform infrared (FTIR) or Raman spectroscopy^28,29^.

The aim of the work described in this study was to investigate permeation rates of vitrification solution components in porcine ovarian tissue. In addition to CPA permeation, water fluxes in the tissue were studied. CPA permeation was studied for vitrification solutions composed of ethylene glycol, dimethyl sulfoxide, glycerol and propylene glycol using three different approaches. Osmometer and differential scanning calorimetry (DSC) measurements were used to study permeation based on the freezing point depression as a function of the CPA exposure duration, whereas FTIR was used to assess permeation based on the appearance of CPA-specific spectral absorbance bands. Tissue dehydration during CPA loading was assessed by weight measurements and correlated with FTIR spectral changes. A novel approach was used to study permeation rates of the solvent compared to that of solutes (CPAs). By using CPA solutions prepared in D_2_O we were able to simultaneously monitor permeation of D_2_O and CPAs and to discriminate between the water flux due to tissue dehydration and water from the CPA solution permeating into the tissue. Diffusion coefficients were derived from experimental data using previously established diffusion models.

## Results

**Figure 1:**
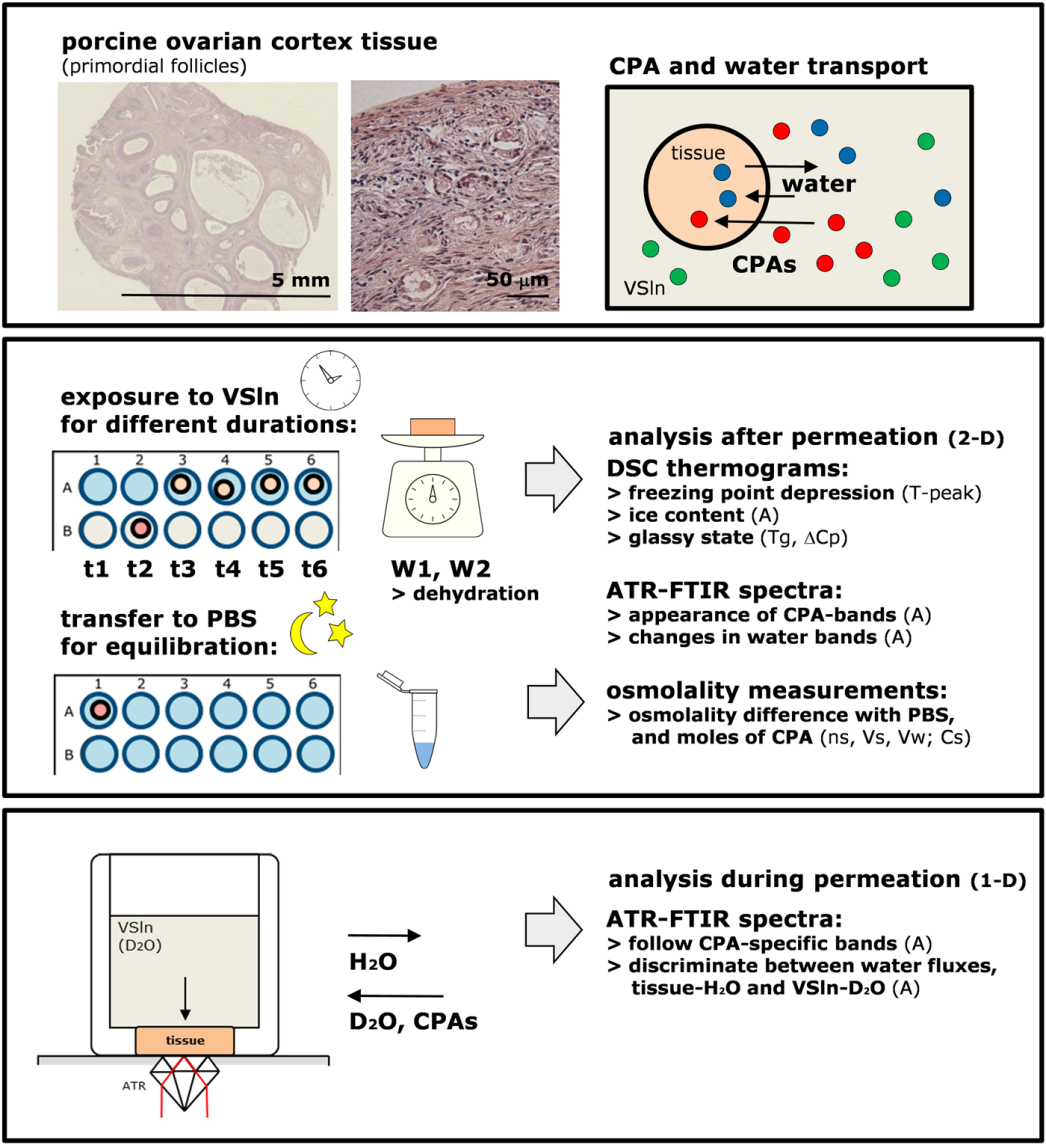
Schematic presentation on sample preparation and different experimental approaches used to study CPA permeation into porcine ovarian tissue. The upper panel shows micrographs of sections of the porcine ovary and cortex tissue, in which follicles of different developmental stages can be recognized (i.e. paraffin embedded material, stained using hematoxylin and eosin). For our measurements, cylindrically shaped tissue punches were cut from the outer cortex region of fresh tissue, just below the germinal epithelium, in which there are primordial (small) follicles. Tissue punches were incubated in vitrification solution (VSln), for analysis of water (blue symbols) and cryoprotective agents (CPAs; red and green symbols) fluxes out and into the tissue. The other panels illustrate the different experimental approaches that were used to study transport processes during CPA loading of ovarian tissue. The middle panel illustrates how tissue pieces were incubated in solutions containing CPAs for various times using 24-well plates (referred to as the 2-D method). Tissue was kept in PBS after their initial weight was determined, and then incubated in VSln for a defined duration after which their weight was assessed again. Thereafter, tissue was either directly analyzed by DSC and FTIR, or transferred into PBS allowing efflux of CPAs that had permeated into the tissue. The latter was quantified by osmometer measurements on the solution after overnight equilibration. The lower panel depicts a schematic design of the ATR-FTIR setup that was used to study CPA and water transport processes during CPA loading of tissues in real time (referred to as the 1-D method). Here VSln was added on top of the tissue, while acquiring IR spectra from the bottom of the tissue. By using VSlns prepared in heavy water (D_2_O) it was possible to discriminate between water in the VSlns and water originating from the tissue (H_2_O).

We previously reported how FTIR can be used to study CPA permeation processes in tissues in real time^28^, which here is referred to as the ‘1-D FTIR method’. Here, we also used a different FTIR approach to study CPA permeation referred to as the ‘2-D FTIR method’ to validate the ‘1-D FTIR method’ and to be able to directly compare FTIR measurements on CPA permeation with osmometer and DSC measurements. In the ‘2-D FTIR method’, sample preparation was done similar as for the osmometer and DSC measurements, and the same model was used to fit the data. Figure 1 shows a schematic presentation of the different experimental approaches that were used here to study CPA permeation and water fluxes that are associated with loading ovarian tissues with cryoprotective agents. Cylindrically shaped tissue punches were used for the diffusion measurements, and transport fluxes were quantified using DSC, osmometer, FTIR, and weight measurements.

### DSC analysis and osmometer measurements to determine CPA permeation and glass formation

**Figure 2:**
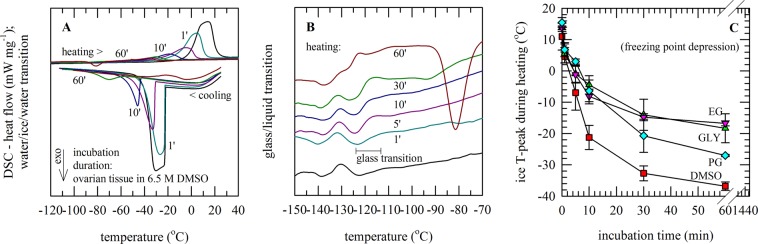
DSC studies on CPA permeation into porcine ovarian tissue. Tissue pieces were incubated for defined durations in solutions containing 6.5 M DMSO (red symbols), GLY (green symbols), EG (pink symbols) or PG (blue symbols). After incubation, tissue was transferred into DSC pans for thermal analysis of the water-to-ice phase transition during cooling and rewarming (**A**) and the glass transition (**B**). CPA permeation into the tissue was determined from the freezing point depression of the ice melting temperature (**C**). Data points reflect mean values with corresponding standard deviations determined from six experiments.

DSC thermograms were collected from ovarian cortex tissue pieces that were incubated in vitrification solutions for different durations. In Fig. 2A it can be seen that the temperature of the peak associated with ice formation decreases with increasing incubation time in vitrification solution (illustrated for DMSO), which coincides with a decrease in the area of the water-to-ice phase transition (i.e. CPA permeation causes freezing point depression). Tissues incubated for 30−60 min in 6.5 M DMSO or PG, exhibited no signs of ice formation in the DSC cooling scans. During warming ice melting is visible as an exothermic event, also for vitrified samples due to devitrification and recrystallization. The temperature of the water-ice phase transition during warming was determined and plotted as a function of the incubation duration (Fig. 2C), to capture differences in CPA permeation kinetics amongst the vitrification solutions. To derive diffusion coefficients, Tm values were normalized towards plateau values determined after 1 h and fitted with Eq. (6) (Table 1).

**Table 1:** Diffusion coefficients of CPAs in ovarian tissue, determined via using different approaches, for single component solutions as well as mixtures.

D (×10^-6^ cm² s^-1^)	2-D			1-D					
	osmometer	DSC	FTIR	FTIR					
	PBS/H_2_O (single)	PBS/H_2_O (single)	PBS/H_2_O (single)	PBS/H_2_O (single)	PBS/H_2_O EG/DMSO	PBS/H_2_O EG/GLY	PBS/H_2_O EG/PG	PBS/D_2_O EG/DMSO	PBS/D_2_O EG/GLY
**EG**	6.04 ± 2.00	7.11 ± 1.25	9.57 ± 0.53	12.17 ± 3.42	10.88 ± 0.49	17.79 ± 1.57	9.83 ± 1.54	11.29 ± 1.09	8.82 ± 0.85
**DMSO**	5.86 ± 1.07	6.91 ± 2.00	8.82 ± 2.11	15.67 ± 5.93	13.06 ± 0.63			13.11 ± 0.80	
**GLY**	5.99 ± 2.24	5.36 ± 0.89	5.73 ± 0.81	6.73 ± 2.10		12.00 ± 2.17			6.79 ± 0.79
**PG**	2.28 ± 0.60	3.93 ± 0.42	4.52 ± 0.97	6.76 ± 0.60			8.59 ± 2.90		

Single component solutions were composed of 6.5 M EG, DMSO, GLY, PG, whereas mixtures contained 3.25 M EG and DMSO, GLY or PG. Osmometer, DSC and ATR-FTIR (2-D) were applied using incubations of tissue in solution, that allowed for CPA movement into the tissue from multiple sites. In addition, ATR-FTIR (1-D) was used to follow CPA permeation through a tissue piece (i.e. in one direction). In the latter case, both ordinary (PBS/H_2_O) and heavy (PBS/D_2_O) buffer/water systems were used for adding CPAs. For all indicated approaches, the respective CPAs for which D-values were determined are indicated in the first column.

For all cases, a glass transition was observed already after 1−5 min incubation of tissue in vitrification solution (illustrated for DMSO in Fig. 2B). The T_g_ was not affected by the incubation duration (DMSO: −122 ± 4 °C, EG: −114 ± 4 °C, PG: −101 ± 3 °C, GLY: −97 ± 4 °C). The change in heat capacity associated with the glass transition during heating, however, increased with increasing incubation duration and CPA content in the tissue. Glass transitions appeared complex, likely due to presence of different (water bound) fractions and occurrence of devitrification with the cooling and warming rates used. After 30−60 min incubation ΔCp values reached a plateau value for all CPAs tested, which were found to be highest for PG and DMSO.

**Figure 3:**
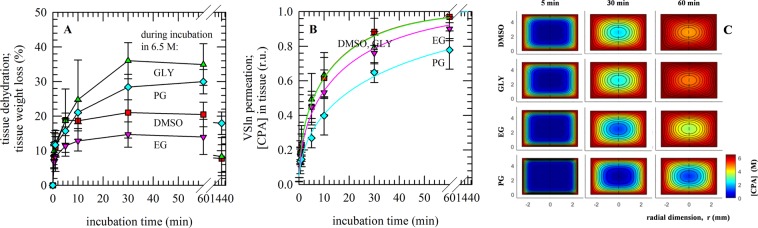
CPA permeation into porcine ovarian tissue determined via osmolality measurements and concomitant tissue dehydration. Tissue pieces were incubated for defined durations in solutions containing 6.5 M DMSO (red symbols), GLY (green symbols), EG (pink symbols) or PG (blue symbols). After incubation for a defined duration in 6.5 M CPA solution, tissue was placed in saline of a known osmolality. Tissue sample weights were determined to assess the extent of tissue dehydration during CPA loading (**A**). Then, after equilibration, medium osmolalities were determined using an osmometer to calculate the tissue CPA concentration (**B**). Mean values with standard deviations have been determined from six experiments. With diffusion coefficients obtained via fitting of the experimental data, the CPA distribution within tissue pieces was modeled at different time points of incubation (**C**).

Osmometer assessment of CPA permeation was done by equilibrating CPA-permeated tissue in saline solution and measuring the freezing point depression caused by the CPAs that have diffused out of the tissue into the saline solution. Quantification was performed by equilibrating the permeated tissue with a surrounding solution and then measuring the osmolality of the solution to determine the amounts of CPAs that have come out of each tissue sample corresponding to each permeation time. Hereby, changes in sample weight due to tissue dehydration were also taken into account (Fig. 3A). Tissue dehydration and weight loss was found to be highest after 1 h exposure to GLY (35 ± 6%), followed by PG (30 ± 4%), DMSO (21 ± 4%) and EG (14 ± 5%). In case of mixtures, exposure to EG/GLY (28 ± 7%) and EG/PG (29 ± 4%) also resulted in a greater weight loss as compared to EG/DMSO (21 ± 3%). In most cases, tissue weights returned close to their original values after about 24 h. It was found that after 30 min tissue CPA contents were highest in solution containing DMSO, followed by GLY, EG and PG. CPA concentrations reached a plateau within one hour. In mixtures, a similar order was found (EG/DMSO > EG/GLY > EG/PG). However, differences were smaller as observed for the single component solutions. In order to fit the data and derive diffusion coefficients, CPA concentration data were normalized towards saturated values determined after 24 h (Fig. 3B), (Table 1). Diffusion coefficients decreased in the order EG, GLY, DMSO > PG. Similar as found with the DSC data, PG permeation was found to be the slowest, whereas permeation rates for DMSO, EG and GLY were found to be similar. Diffusion coefficients that were derived for the CPAs were used to calculate and visualize differences in CPA distribution in tissue pieces of a defined geometry as a function of the incubation time in (Fig. 3C).

### ATR-FTIR analysis of CPA permeation and tissue dehydration

**Figure 4:**
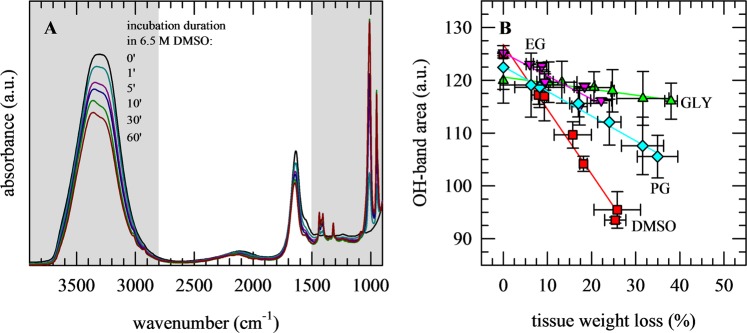
ATR-FTIR analysis of CPA permeation and concomitant dehydration of porcine ovarian tissue during incubation in vitrification solution. Tissue pieces were incubated for defined durations in solutions containing 6.5 M DMSO (red symbols), GLY (green symbols), EG (pink symbols) or PG (blue symbols), after which spectra were collected. CPA permeation is evident as appearance of specific absorbance bands in the finger print region ranging from 1500–900 cm^−1^. The 3800–2800 cm^−1^ spectral region shows changes in the OH-stretching vibration band due to tissue dehydration during incubation in CPA solution (**A**). The decrease in the water band area was correlated with the tissue weight loss (**B**). Mean values with standard deviations are determined from triplicate measurements.

Using a similar approach as described above for the osmometer and DSC measurements, ovarian tissue pieces were incubated in CPA solutions, after which tissue pieces were collected and ATR-FTIR spectra were collected as a function of the incubation time. Upon incubation, a decrease in the OH-stretching vibration band (νOH at ~3300 cm^−1^), predominantly arising from water, was observed coinciding with the appearance of CPA-specific bands in the 1500−900 cm^−1^ spectral region (Fig. 4A). The relative increase of CPA-specific band areas was determined, and plotted as a function of the incubation time. The data were fitted using Eq. 6 to derive diffusion coefficients. This is referred to as the 1-D FTIR method in Table 1.

The area of νOH was found to correlate with the extent of tissue dehydration (weight loss) due to CPA exposure for all CPAs (Fig. 4B). The differences in slopes in this plot are not necessarily related to differences in tissue dehydration, but can be attributed to differences in contributions of CPA OH groups to the OH stretching band. In addition to water, also OH-groups CPAs may contribute to the OH stretching band. At the same dehydration level, the area of the OH band remains relatively high in case of glycerol because of its OH groups contributing to this band area. Likewise, the lack of OH groups in DMSO result in a relatively low OH stretching band area compared to that of e.g. glycerol at the same tissue dehydration level.

**Figure 5:**
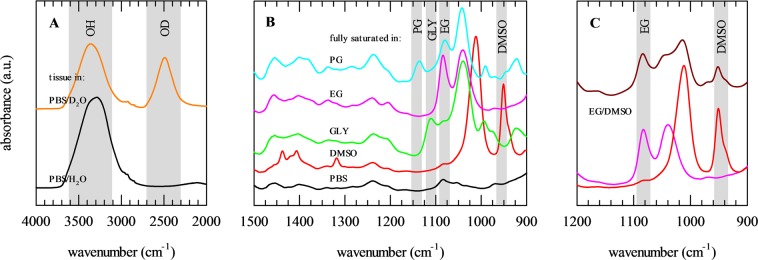
Characterization of CPA-specific infrared absorbance bands in porcine ovarian tissue saturated with various vitrification solutions. Tissue pieces were incubated in solution containing DMSO (red lines), GLY (green lines), EG (pink lines) or PG (blue lines), as well as mixtures thereof (EG/DMSO; brown lines). CPA solutions were prepared in PBS; while using ordinary (PBS/H_2_O; black lines) or deuterated (PBS/D_2_O; orange lines) water. The OH and OD-stretching vibration bands can be found in the 4000–2000 cm^−1^ spectral region, whose intensities respectively decrease and increase with dehydration and permeation (**A**). CPA-specific bands can be found in the 1500–900 cm^−1^ spectral region (**B**). Spectra of fully saturated tissue were used to assign CPA-specific wavenumber regions with minimal overlap (indicated in grey), which allowed for discrimination in mixtures (**C**).

To obtain more insights in the different water fluxes associated with CPA loading, heavy water was used. This allowed discriminating between νOH originating from water in the tissue itself and νOD (~2500 cm^−1^) from heavy water in the vitrification solution permeating into the tissue (Fig. 5A). CPA permeation into the tissue can be monitored using the characteristic spectral fingerprint of each CPA. In Fig. 5B, the 1500–900 cm^−1^ spectral region is shown for tissue saturated with vitrification solution illustrating specific wavenumber ranges for each of the CPAs (i.e. DMSO, GLY, EG and PG). Spectral ranges for each of the CPAs were selected that exhibited minimal overlap in mixtures (Fig. 5C).

### Simultaneous monitoring multiple CPA components in mixtures using ATR-FTIR

**Figure 6:**
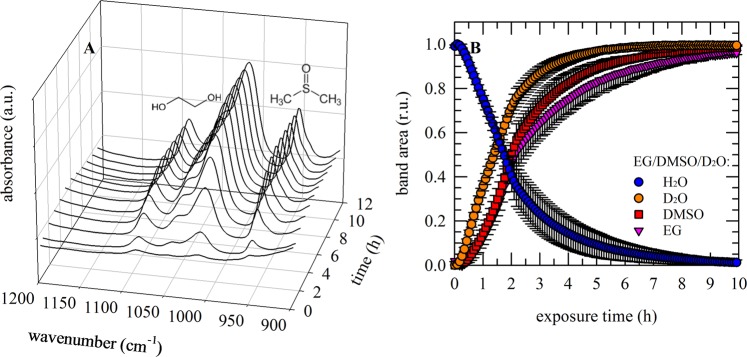
Simultaneous monitoring of multiple CPAs permeating into porcine ovarian tissue and concomitant water fluxes, using ATR-FTIR. Tissue was mounted in a holder on the ATR-crystal, vitrification solution was added on top and spectra were acquired during permeation through the tissue (**A**; EG/DMSO). Movement of CPA through the tissue was evident as the appearance of specific absorbance bands, whose area were determined and their relative increase was plotted versus time (DMSO: red symbols, EG: pink symbols). In addition, since the CPA solution was prepared in PBS/D_2_O, it was possible to follow the decrease and increase in respectively the OH- (blue symbols) and OD- (orange symbols) stretching vibration bands, due to tissue dehydration and permeation of the CPA solution. In order compare kinetics, band areas were normalized to values obtained after 16−24 h. Mean values with standard deviations have been determined from triplicate measurements.

In order to monitor diffusion or permeation in real time, a setup was used where solution containing CPAs was added on top of a tissue sample while collecting ATR-FTIR spectra at the bottom of the sample (in contact with the ATR crystal) as a function of time (Fig. 6A). Movement of CPAs and water through the tissue is evident as appearance of specific absorbance bands, whose area can be calculated and plotted versus time. For a mixture of EG/DMSO in PBS/D_2_O, both the decrease and increase of respectively νOH and νOD can be followed as well as appearance of bands specific for EG and DMSO. In order to compare kinetics, band areas were normalized to values obtained after 16–24 h. In Fig. 6B it can be seen that the decrease in νOD band area (D_2_O permeation), occurs faster compared to the decrease in νOH (mainly due to tissue dehydration). Again, it should be noted that also CPA OH groups contribute to the νOH band, whereas the νOD band solely originates from D_2_O. In any case, (heavy) water movement occurs faster compared to CPA permeation, which likely can be attributed to the relatively small size of water. Furthermore, it can be seen that DMSO permeates faster than EG when tissue is exposed to a DMSO/EG mixture.

**Figure 7:**
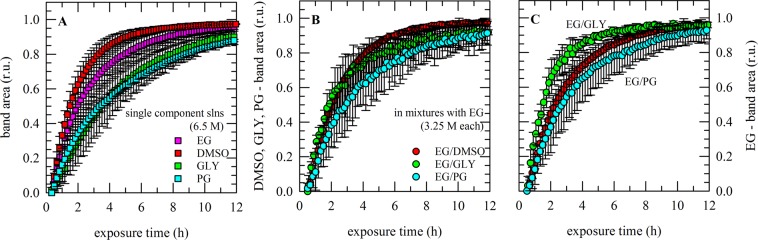
CPA permeation into porcine ovarian tissue, determined in real time via ATR-FTIR. Tissue was mounted in a holder on the ATR-crystal, vitrification solution was added on top and spectra were acquired during permeation through the tissue. Both single component solutions (**A**) as well as mixtures (**B**,**C**) were tested. Single component solutions were composed of 6.5 M DMSO (blue symbols), GLY (red symbols), EG (green symbols) or PG (purple symbols). Mixtures contained 3.25 M EG and 3.25 M DMSO, GLY or PG. The relative increase in CPA-specific band areas was determined (i.e. values were normalized to those obtained after 16−24 h) and plotted as a function of the exposure time (**A**–**C**). Average curves with standard deviations are presented, determined from triplicate measurements.

With single component solutions, clear differences in permeation rates were observed among the different CPAs tested (Fig. 7A). The relative increase in CPA band area was fastest for DMSO, followed by EG and then GLY and PG. Maximum values (i.e. full saturation) were reached after approximately 10 and 12 h for DMSO and EG, respectively, whereas for GLY and PG much longer incubation times were needed to reach saturation (~16 h). As illustrated in Fig. 5, CPA-specific absorbance bands were also analyzed in mixtures. Also in mixtures, permeation of EG and DMSO was found to be fastest, whereas PG permeation was slowest (Fig. 7B). Of note is that EG permeation rates are quite different in the different mixtures; EG diffusion is fastest in a mixture with GLY, and slowest in a mixture with PG (Fig. 7C). Plots were fitted using Eq. (7) to derive diffusion coefficients. This is referred to as the 2-D FTIR method in Table 1. D-values obtained using the 2-D method are greater than those obtained using the 1-D method.

## Discussion

Vitrification of ovarian tissues relies on the use of protective solutions to avoid ice formation during cooling and warming. Vitrification solutions are typically composed of mixtures of high concentrations of different permeating agents (i.e. CPAs), which need to be introduced into the tissue prior to cooling. The tissue matrix and cells actually form a barrier for CPAs to quickly enter into the tissue. When tissue is immersed in a vitrification solution; osmotic and concentration gradient driving forces result in CPA and water transport fluxes, until equilibrium state is reached. Understanding and quantifying these transport processes allows rationally designing protocols for loading tissues with CPAs for cryopreservation (i.e. minimizing osmotic excursions, exposure durations and toxicity effects). In the current study, we used different spectroscopic and thermal approaches for visualizing, identifying and quantifying transport processes taking place when porcine ovarian cortex tissues are exposed to solutions containing CPAs.

First, we studied tissue incubated in vitrification solutions for different durations, referred to as 2-D measurements in Table 1, in which CPAs can enter the tissue from all sites. With FTIR, tissue CPA permeation was assessed based on spectral fingerprinting as a function of the incubation time. When tissue was analyzed using DSC it was found that incubation and permeation with CPAs coincides with a decrease in the tissue freezing and melting temperature, as well as appearance of a glassy state when the tissue is cooled. This reflects the replacement of water by CPAs in the tissue. To the best of our knowledge, the use of DSC to study CPA permeation in tissues provides an additional experimental approach to validate CPA permeation data obtained with other methods. DSC not only can be used to derive CPA permeation rates, but also provides insights in glass formation during CPA permeation. It is shown that the change in heat capacity associated with the glass transition increased with increasing CPA content in the tissue. Osmolality measurements are also based on freezing point depression. However, in this case dilute solutions were analyzed in which CPA-permeated tissue was equilibrated. All these methodologies revealed that DMSO permeates fastest into ovarian tissue followed by PG, GLY and EG, and derived diffusion coefficients closely match. Using a different FTIR approach, referred to as 1-D measurements in Table 1, diffusion can be studied in ‘real time’ on the same sample without further manipulation^28,30^. Comparative diffusion coefficients derived using this approach were higher. This may be explained by the fact that samples are positioned in a sample holder with permeating solution added on top of the sample, and not freely floating in solution as is the case with the other approaches. In addition, different models were used to fit the data.

Compared to previous reports with other tissue types, the CPA diffusion coefficients determined in this study for ovarian tissue are slightly higher but in the same order of magnitude. For example, DMSO diffusion in cartilage tissue analyzed using osmometer measurements was found to have a D-value of 3.5 × 10^−6^ cm^2^ s^−1^ ^27^, whereas CT measurements showed that the diffusion coefficient of DMSO in collagen scaffolds is 2.4 × 10^−6^ cm^2^ s^−1^ at 20 °C^31^. Furthermore, decellularized heart valve tissues, analyzed using FTIR and the 1-D approach, exhibited a D-value of 3.02 × 10^−6^ cm^2^ s^−1^ for DMSO^28^. In addition to the incubation temperature, diffusion rates of CPAs in tissue depend on their molecular weight^30^, as well as chemical properties. According to the Stokes-Einstein equation diffusion is inversely related to viscosity. Based on its lower viscosity and smaller molecular size it can be expected that EG permeates faster than GLY. This is indeed observed in the FTIR methods (almost factor 2 difference), whereas in the other 2 methods the difference between EG and GLY is much smaller. In addition, using FTIR, we were also able to determine differences in diffusion behavior dependent on the mixture composition. Differences in diffusivity observed in multi-component mixtures are explained by interactions between the components of the mixture^32^. It should be noted that Fick’s law for diffusion of a single solute is not applicable for two solutes that diffuse at different rates. So, in case of mixtures the D-values that are reported here should be interpreted as phenomenological parameter describing the rate at which solutes enter the tissue.

Exposing tissue to vitrification solution was found to cause severe weight losses, which may be related to tissue matrix as well as cellular dehydration. The initial tissue weight loss was strongest with GLY and least with EG. This can possibly be explained by the fact that, among the CPAs that were tested, GLY is the least membrane permeable^17^. The model applied in our studies to derive D-values did not take into account effects caused by cells in the tissue. More complex models are available that also include biomechanical forces^22,33^. Tissue dehydration coincided with a decrease of the OH-stretching vibration band, suggesting this band can be used as a spectroscopic marker for tissue dehydration. Experiments using vitrification solutions that were prepared with heavy water, showed that the increase in the OD band area (i.e. water permeation into the tissue) occurred slightly faster compared to the decrease in OH band area (i.e. tissue dehydration). Both processes, however, are faster compared to CPA permeation, indicating water moves faster through the tissue as CPAs.

Taken together, diffusion coefficients derived from FTIR studies closely match those obtained by DSC and osmometer measurements. FTIR allows for the most detailed assessment of the transport processes taking place; different solutes in mixtures can be discriminated and monitored during CPA loading of tissue, while also concomitant water fluxes associated with tissue dehydration and water permeation can be monitored.

## Materials and Methods

### Vitrification solutions and preparation of porcine ovarian tissue pieces

Various vitrification solutions were tested, including: 6.5 M ethylene glycol (EG; 36% v/v), dimethylsulfoxide (DMSO; 46% v/v), glycerol (GLY; 48% v/v) and propylene glycol (PG; 49% v/v). In addition, mixtures of two components: EG/DMSO, EG/GLY, EG/PG, containing 3.25 M of each component, were studied. All solutions were prepared in phosphate buffered saline (PBS, pH 7.4), with final concentrations of: 137 mM NaCl, 27 mM KCl, 10 mM Na_2_HPO_4_, 1.8 mM KH_2_PO_4_. This was done using a 4 × PBS stock solution. For some of the experiments, heavy/deuterated water (D_2_O) was used as diluent instead of ordinary water (H_2_O).

Ovaries were collected from pre-pubertal gilts at a local slaughterhouse, and transported to the lab within 1−2 h in an insulated container. Upon arrival the temperature was checked (30−33 °C) and ovaries were rinsed with 0.9% (w/v) NaCl, followed by cooling to room temperature. Cylindrically shaped tissue punches (5 mm height and diameter) were cut from the outer cortex region, just below the germinal epithelium. Large follicles and ovarian ligament were avoided. Figure 1 (upper panel) shows images of ovarian cortex tissue. Tissue pieces were stored in PBS at 4 °C and used within 24 h. Tissue pieces had a weight of 45 ± 10 mg, and the water content was 90 ± 3% of the fresh weight. Dry weights were determined after 24 h evaporation of water in an incubator set at 80 °C.

### CPA diffusion kinetics determined using osmolality measurements

Osmometer measurements were performed to determine permeation of specific CPAs into tissue as previously described^26,27^, with minor modifications (Fig. 1). Solution densities were measured using a density meter (DMA38; Anton Paar GmbH, Graz, Austria). Incubations were done at room temperature (~22 °C). Ovarian tissue pieces were handled singly using 24-well tissue culture plates, containing 2 mL solution per well. First, tissue pieces were maintained in PBS to determine their initial weight (w_1_). Weights were determined after removal of excess solution by gentle dipping on paper towel. Then, tissue was immersed in vitrification solution for various durations (0, 0.5, 1, 5, 10, 30, 60 min, and 24 h), after which weights were determined again (w_2_). Thereafter, CPA-perfused tissue pieces were transferred into fresh PBS for equilibration (i.e. allow release of CPA into PBS) during 24 h incubation at 4 °C. After equilibration, the osmolality of the CPA/PBS-solution was measured using a freezing point depression osmometer (OM801; Vogel, Fernwald, Germany). Each treatment was repeated six times, using separate tissue pieces originating from at least three different collections of ovaries.

The extent of tissue dehydration was calculated by comparing weights before and after incubation in vitrification solution:

1 $${W}_{loss}( \% )=\frac{{W}_{1}(g)-{W}_{2}(g)}{{W}_{1}(g)}\times 100( \% )$$

To determine the tissue CPA content as a function of the incubation time, the osmolality of PBS itself (π_PBS_) was compared with the osmolality determined after equilibrating permeated tissue in PBS (π_s_). Therewith the CPA content (n_s_, in moles) in the surrounding solution (V: 2 mL, ρ: 1.0 g mL^−1^) was determined:

2 $${n}_{s}(mol)=[{\pi }_{s}-{\pi }_{PBS}](\frac{mmol}{kg})\times \frac{1}{1000}(\frac{mol}{mmol})\times \frac{1}{1000}(\frac{kg}{g})\times V(mL)\times \rho (\frac{g}{mL})$$

It is assumed that the amount of CPA remaining in the tissue after equilibration can be ignored. Using the CPA molar mass (MW_s_) and solution density (ρ_s_: 1.1 g mL^−1^) the CPA mass and volume permeated into the tissue can be calculated (V_s_). In addition, the water volume can be calculated (V_w_), taken into account the density of water at room temperature (ρ; 1.0 g mL^−1^).

3 $${V}_{s}(mL)=\frac{{n}_{s}(mol)\times M{W}_{s}(\frac{g}{mol})}{{\rho }_{s}(\frac{g}{mL})}$$

4 $${V}_{w}(mL)=\frac{{w}_{2}(g)-[{f}_{dm}\times {w}_{1}(g)+{n}_{s}(mol)\times M{W}_{s}(\frac{g}{mol})\,]}{\rho (\frac{g}{mL})}$$

here f_dm_×w_1_ represents the tissue dry matter, with f_dm_ equals 0.1 since the tissue water content was 90%. Now, the tissue CPA concentration (C_s_) can be calculated as follows:

5 $${C}_{s}(\frac{mol}{L})=\frac{{n}_{s}(mol)}{{V}_{s}(mL)+{V}_{w}(mL)}\times 1000(\frac{mL}{L})$$

### Data fitting to derive diffusion coefficients

Plots in which the CPA concentration was plotted as a function of the incubation time in vitrification solution were fitted to derive diffusion coefficients, using MATLAB software (Mathworks, Natick, MA, USA). A model based on Fick’s second law of diffusion was used to describe diffusion into a cylindrically shaped body. As previously described^27,34^, the numerical solution of the model describing two-dimensional diffusion was converted to a one-dimensional solution, by multiplying the one-dimensional solutions for an infinite slab and cylinder:

6 $$\frac{{C}_{s}-{C}_{s}^{\ast }}{{C}_{s0}-{C}_{s}^{\ast }}=[\frac{8}{{\pi }^{2}}{\sum }_{n=0}^{\infty }\frac{1}{{(2n+1)}^{2}}exp(\frac{-D{(2n+1)}^{2}{\pi }^{2}t}{4{a}^{2}})]\times [\frac{4}{{R}^{2}}{\sum }_{n=1}^{\infty }\frac{1}{{b}_{n}^{2}}exp(-D{b}_{n}^{2}t)]$$

here C_s0_ represents the initial average CPA concentration (in mol L^−1^), C_s_ the concentration after incubation for a specific duration (t in s), and C_s_^*^ the assumed final concentration which was taken as boundary condition (6.5 mol L^−1^ for single component solutions). Furthermore, ‘a’ represents half the thickness of the axial dimension (0.25 cm; the tissue was exposed to CPAs from both sides), R is the radius of the cylinder (0.25 cm), D is the diffusion coefficient (in cm^2^ s^−1^), and b_n_’s are the roots of the zero-order Bessel function of the first kind with J_0_(b_n_R) equals 0.

### Differential scanning calorimetry (DSC)

DSC thermograms were collected using a Netzsch 204F1 Phoenix instrument (Netzsch-Geraetebau GmbH, Selb, Germany). Calibration was performed according to the instructions provided by the manufacturer, and an empty pan was used as a reference sample. Ovarian tissue pieces were incubated in vitrification solutions for different durations in 24-well culture plates as described above (Fig. 1). In contrast to the other incubations, samples of a smaller thickness (0.2 cm) were prepared (27 ± 6 mg) that fit in the 25-μL aluminum sample pans. Samples were cooled down to −150 °C with 20 °C min^−1^ followed by heating to 30 °C at 10 °C min^−1^, while monitoring the heat flow. Thermograms were analyzed using Netzsch software. Each treatment was repeated three times using separate tissue pieces originating from three different collections of ovaries.

CPA diffusion into the tissue was monitored by following freezing point depression and the gradual disappearance of the ice melting peak. In addition, glass transitions were derived from the traces. Plots of the ice melting temperature (T_m_-peak) and content (peak-area, in J g^−1^) as a function of the incubation duration in vitrification solution were constructed. Similar as with the osmometer measurements, it was assumed that T_m_ decreases linearly with increasing solute concentration, and hence is proportional to the CPA concentration in the tissue. In order to estimate diffusion coefficients, Eq. (6) with ‘a’ 0.1 cm was used to fit the experimental data. The appearance of a glassy state in the tissue samples during incubation was monitored by looking at the glass transition temperature (T_g_-midpoint) and specific heat capacity (ΔC_p_, in J g^−1^ K^−1^).

### Fourier-transform infrared (FTIR) spectroscopy

Infrared spectra were recorded using a PerkinElmer 100 FTIR spectrometer (PerkinElmer, Waltham, MA, USA), equipped with a triglycine sulfate detector and an attenuated total reflection (ATR) accessory with a 1 × 1 mm^2^ diamond/ZnSe crystal. Spectra acquisition parameters were: 4 cm^−1^ resolution, 8 co-added interferograms and a 4000–650 cm^−1^ wavenumber range. An automatic CO_2_/H_2_O vapor correction algorithm was used during recording of the spectra. Spectral analysis was done using Omnic software (Thermo Fisher Scientific, Waltham, MA, USA).

Two approaches were used to measure CPA diffusion with ATR-FTIR (Fig. 1): (i) tissue pieces were incubated in vitrification solutions for different durations, in 24-well culture plates, after which infrared spectra were collected, and (ii) tissue pieces were mounted in a holder positioned on the ATR crystal that allowed adding vitrification solution on top of the sample, while monitoring diffusion through the tissue by collecting spectra every 10 min over a period of 18–24 h. In both cases, measurements were done at room temperature and repeated three times.

Saturated tissue was used to select specific infrared absorbance bands of EG (1110–1064 cm^−1^), DMSO (966–921 cm^−1^), GLY (1137–1106 cm^−1^) and PG (1154–1120 cm^−1^) that had no or minimal overlap with each other. The relative increase in the areas of CPA-specific absorbance bands was determined as a function of the exposure time. The spectrum of tissue without CPA permeation was subtracted from that taken at a given time point, the band area in the indicated wavenumber ranges was calculated, and normalized towards the value determined after 18–24 h diffusion (i.e. fully saturated tissue). In case incubations were done in 24-well plates (method i), tissue samples were mounted using the ATR force arm using only little pressure on the sample to facilitate contact with the ATR crystal, and data were fitted using Eq. (6). Tissue dehydration was studied by looking at the decrease in the OH-stretching vibration band (3700–3000 cm^−1^), and was compared with tissue weight loss measurements performed on the same samples. In addition, D_2_O was used as diluent to detect water movement into the tissue based on the increase in the OD-stretching vibration band (2800–2100 cm^−1^).

### Data fitting data with continuous FTIR diffusion measurements

For real-time measurements on CPA diffusion or permeation (method ii), tissue punches were mounted in a holder on the ATR crystal as previously described in detail^28,30^. This setup allowed adding 1 mL vitrification solution on top of a cylindrically shaped tissue piece with a 5 mm diameter and height, while monitoring CPA movement through the tissue as the appearance of specific absorbance bands.

The diffusion process was modeled using Fick’s law of diffusion for a one-dimensional flow into a film, as previously described^35^. The absorbance at a given time point (A_t_) normalized towards the absorbance at equilibrium (A_∞_) was fitted using MATLAB and the following equation:

7 $$\frac{{A}_{t}}{{A}_{\infty }}=1-\frac{\frac{8\gamma }{\pi (1-exp(-2\gamma L))}{\sum }_{n=0}^{\infty }\exp (-\frac{D{(2n+1)}^{2}{\pi }^{2}t}{4{L}^{2}})[{(-1)}^{n}2\gamma +\frac{(2n+1)\pi }{2L}\exp (-2\gamma L)]}{(2n+1)[4{\gamma }^{2}+{(\frac{(2n+1)\pi }{2L})}^{2}]}$$

where L is the thickness or height of the tissue (0.5 cm; since the tissue was exposed to CPAs from one side), t is time (in s) and D is the diffusion coefficient (in cm^2^ s^−1^). γ is the evanescent decay coefficient, which is defined as the inverse of the penetration depth (d_р_; in cm):

8 $$\gamma =\frac{1}{{d}_{p}}=\frac{2{n}_{1}\pi \sqrt{{(\sin \theta )}^{2}-{(\frac{{n}_{2}}{{n}_{1}})}^{2}}}{\lambda }$$

here n_1_ and n_2_ are the refractive index values of the ATR crystal and tissue (2.43 and 1.4, respectively), θ is the incident angle of the infrared rays (45°), and λ the inverse of the wavenumber position with maximum absorbance (1085 cm^−1^ for EG, 951 cm^−1^ for DMSO, 1111 cm^−1^ for GLY, 1136 cm^−1^ for PG).

## References

[1] Sonmezer M, Oktay K (2004). Fertility preservation in female patients. Hum. Reprod. Update.

[2] Donnez J, Dolmans MM (2017). Fertility preservation in women. New Engl. J. Med..

[3] Fisch B, Abir R (2018). Female fertility preservation: past, present and future. Reproduction.

[4] Gosden RG, Baird DT, Wade JC, Webb R (1994). Restoration of fertility to oophorectimized sheep by ovarian autografts stored at −196 °C. Hum. Reprod..

[5] Meirow D (2005). Pregnancy after transplantation of cryopreserved ovarian tissue in a patient with ovarian failure after chemotherapy. New Engl. J. Med..

[6] Donnez J (2006). Ovarian tissue cryopreservation and transplantation: a review. Hum. Reprod. Update.

[7] Silber SJ (2012). Ovary cryopreservation and transplantation for fertility preservation. Mol. Hum. Reprod..

[8] Picton HM, Harris SE, Muruvi W, Chambers EL (2018). The *in vitro* growth and maturation of follicles. Reproduction.

[9] Liebenthron J (2013). The impact of culture conditions on early follicle recruitment and growth from human ovarian cortex biopsies *in vitro*. Fert. Steril..

[10] Bulgarelli DL, Ting AY, Gordon BJ, de Sá Rosa-e-Silva ACJ, Zelinski MB (2018). Development of macaque secondary follicles exposed to neutral red prior to 3-dimensional culture. J. Assist. Reprod. Genet..

[11] Santos RR (2010). Cryopreservation of ovarian tissue: an emerging technology for female germline preservation of endangered species and breeds. Anim. Reprod. Sci..

[12] Keros V (2009). Vitrification versus controlled-rate freezing in cryopreservation of human ovarian tissue. Hum. Reprod..

[13] Ting AY, Yeoman RR, Lawson MS, Zelinski MB (2011). *In vitro* development of secondary follicles from cryopreserved rhesus macaque ovarian tissue after slow-rate freeze or vitrification. Hum. Reprod..

[14] Mazur P (1984). Freezing of living cells: mechanisms and implications. Am. J. Physiol..

[15] Mazur, P. Principles of cryobiology. In: *Life in the frozen state* (ed. Fuller, B. J., Lane, N. & Bensson, E. E.) 3−65 (CRC Press). ISBN 0-415-24700-4 (2004)

[16] Pegg, D. Principles of cryopreservation. In: *Cryopreservation and freeze-drying protocols. Methods in Molecular Biology* (eds Wolkers, W. F. & Oldenhof, H.) 1257, 3–19 (Springer, 2015). 10.1007/978-1-0716-0783-1_1 32797407

[17] Wolkers WF (2019). Factors affecting the membrane permeability barrier function of cells during preservation technologies. Langmuir.

[18] Fahy GM, MacFarlane DR, Angell CA, Meryman HT (1984). Vitrification as an approach to cryopreservation. Cryobiology.

[19] Fahy, G. M. & Wowk, B. Principles of cryopreservation by vitrification. In: *Cryopreservation and freeze-drying protocols. Methods in Molecular Biology* (ed. Wolkers, W. F. & Oldenhof, H.) 1257, 21–82 (Springer, 2015). 10.1007/978-1-4939-2193-5_2 25428002

[20] Benson JD, Higgins AZ, Desai K, Eroglu A (2017). A toxicity cost function approach to optimal CPA equilibration in tissues. Cryobiology.

[21] Elmoazzen HY (2007). Dimethyl sulfoxide toxicity kinetics in intact articular cartilage. Cell Tissue Bank..

[22] Shaozhi Z, Pegg DE (2007). Analysis of the permeation of cryoprotectants in cartilage. Cryobiology.

[23] Abazari A, Elliott JAW, McGann LE, Thompson RB (2012). MR spectroscopy measurement of the diffusion of dimethyl sulfoxide in articular cartilage and comparison to theoretical predictions. Osteoarthritis and Cartilage.

[24] Bischof JC, Mahr B, Choi JH, Behling M, Mewes D (2007). Use of X-ray tomography to map crystalline and amorphous phases in frozen biomaterials. Ann. Biomed. Eng..

[25] Corral A (2015). Assessment of the cryoprotectant concentration inside a bulky organ for cryopreservation using X-ray computed tomography. Cryobiology.

[26] Sharma R (2007). A novel method to measurure cryoprotectant permeation into intact articular cartilage. Cryobiology.

[27] Jomha NM (2009). Permeation of several cryoprotectant agents into porcine articular cartilage. Cryobiology.

[28] Vásquez-Rivera A (2018). Simultaneous monitoring of different vitrification solution components permeating into tissues. Analyst.

[29] Marzi J (2019). Marker-independent *in situ* quantitative assessment of residual cryoprotectants in cardiac tissues. Anal. Chem..

[30] Wang S (2014). Protein stability in stored decellularized heart valve scaffolds and diffusion kinetics of protective molecules. Biochim. Biophys. Acta.

[31] Bernemann I (2010). Diffusion of dimethyl sulfoxide in tissue engineered collagen scaffolds visualized by computer tomography. Cryo Letters.

[32] Krishna R, Wesselingh JA (1997). The Maxwell-Stefan approach to mass transfer. Chem. Eng. Sci..

[33] Abazari A, Thompson RB, Elliott JA, McGann LE (2012). Transport phenomena in articular cartilage cryopreservation as predicted by the modified triphasic model and the effect of natural inhomogeneities. Biophys. J..

[34] Abazari A, Jomha NM, Law GK, Elliott JAW, McGann LE (2009). Erratum to ‘Permeation of several cryoprotectant agents into porcine articular cartilage [Cryobiology 58 (2009) 110–114]’. Cryobiology.

[35] Fieldson GT, Barbari TA (1995). Analysis of diffusion in polymers using evanescent field spectroscopy. AIChE J..

